# Effortful Eyelid Opening With Enhanced Mechanoreceptor Stretch in the Superior Tarsal Muscle Accelerates Inferred Microsaccade Frequency and Velocity, Exacerbating Reflexive Frequent Blinking: A Report of Two Cases

**DOI:** 10.7759/cureus.117769

**Published:** 2026-10-11

**Authors:** Kiyoshi Matsuo, Ai Kaneko

**Affiliations:** 1 Plastic Surgery Oculoplastic Surgery, Matsuo Plastic and Oculoplastic Surgery Clinic, Hamamatsu, JPN; 2 Plastic and Reconstructive Surgery, Shinshu University School of Medicine, Matsumoto, JPN; 3 Plastic and Oculoplastic Surgery, Matsuo Plastic and Oculoplastic Surgery Clinic, Hamamatsu, JPN; 4 Plastic and Reconstructive Surgery, Hamamatsu University School of Medicine, Hamamatsu, JPN

**Keywords:** global layer of inferior rectus muscle, global layer of superior rectus muscle, inferior tarsal muscle, levator palpebrae superioris muscle, mesencephalic trigeminal nucleus, microsaccades frequency and velocity, rostral interstitial nucleus of medial longitudinal fasciculus, rostral locus coeruleus, rostral superior colliculus, superior tarsal muscle

## Abstract

To maintain eyelid opening and arousal without proprioceptive or visual fading, microsaccades of fast-twitch fibers (FTFs) in the levator palpebrae superioris muscle (LPSM) and the global layer of the superior rectus muscle (GLSRM) continually stretch mechanoreceptors in the superior tarsal muscle (STM). This stretch generates proprioceptive signals that induce reflex contractions of LPSM slow-twitch fibers (STFs) and regulate arousal via the mesencephalic trigeminal nucleus (MTN) and locus coeruleus (LC). Concurrently, counteracting microsaccades of FTFs in the global layer of the inferior rectus muscle (GLIRM) stretch putative mechanoreceptors in the inferior tarsal muscle (ITM), inducing reflex contractions of STFs in the palpebral orbicularis oculi muscle (POOM) that anchor the eyelid to the globe and, when excessive, can produce apraxia of eyelid opening. We previously reported a patient with right lower eyelid retractor (LER) disinsertion who had sensitized right ITM mechanoreceptors. She could voluntarily provoke clonic reflex contractions of the right POOM STFs by combining maximal downgaze (effortful GLIRM FTF contractions that enhance ITM stretch) with maximal bilateral upper eyelid opening (effortful LPSM and GLSRM FTF contractions that further enhance STM stretch). This indicated that the contractions resulted from additive overstimulation of STM mechanoreceptors rather than ITM stretch alone. Because the superior colliculus (SC) generates microsaccades via the LC and elevated arousal increases microsaccadic velocity, we hypothesized that enhanced STM mechanoreceptor stretch raises arousal and accelerates the frequency and velocity of microsaccades in LPSM, GLSRM, and GLIRM FTFs via the MTN, LC, and SC. Accelerated microsaccades would continually restretch STM or ITM mechanoreceptors, inducing clonic reflex contractions of LPSM or POOM STFs that manifest as frequent blinking (FB). To test this hypothesis, we examined two patients with bilateral disinsertions of the levator aponeurosis and LER. Their sensitized STM and ITM mechanoreceptors were expected to provoke FB during effortful eyelid opening. We modulated STM and ITM stretch by loading a 3 g weight on the upper eyelid and by digitally elevating or pulling down the lower eyelid, then quantified blink dynamics by video analysis. Preoperatively, digital elevation of the lower eyelid (enhancing ITM stretch) increased reflex POOM STF contractions. These contractions antagonized upper eyelid opening, further stretched the STM, increased reflex LPSM STF contractions, and accelerated FB frequency and velocity in a degree-dependent manner. Adding the 3 g upper eyelid weight further accelerated FB dynamics during effortful opening, whereas pulling down the lower eyelid (slackening the ITM) reduced POOM reflex contractions, slackened the STM, and abolished FB. After surgical aponeurosis reinsertion restored tarsal stability and reduced baseline STM stretch, the combination of upper eyelid weight loading and lower eyelid elevation no longer induced FB at the two-week or six-month follow-up. These clinical observations suggest that enhanced STM mechanoreceptor stretch may accelerate inferred FTF microsaccades across the LPSM, GLSRM, and GLIRM, thereby driving reflex contractions of the antagonistic LPSM and POOM STFs and exacerbating FB.

## Introduction

To maintain eyelid opening [1,2] and arousal [3-6] without proprioceptive or visual fading [7], microsaccades of fast-twitch fibers (FTFs) in the levator palpebrae superioris muscle (LPSM) and the global layer of the superior rectus muscle (GLSRM) [8] continually stretch mechanoreceptors in the superior tarsal muscle (STM) [9]. This stretch elicits proprioceptive signals that induce reflex contractions of slow-twitch fibers (STFs) in the LPSM [1,2] and regulate arousal [3-6] via the mesencephalic trigeminal nucleus (MTN) and rostral locus coeruleus (RLC) [10]. Concurrently, counteracting microsaccades of FTFs in the global layer of the inferior rectus muscle (GLIRM) stretch putative mechanoreceptors in the inferior tarsal muscle (ITM), inducing reflex contractions of STFs in the palpebral orbicularis oculi muscle (POOM) that anchor the eyelid to the globe; when excessive, these contractions can produce apraxia of eyelid opening [11]. We previously reported a patient with right lower eyelid retractor (LER) disinsertion who had sensitized right ITM mechanoreceptors [11]. She could voluntarily provoke clonic reflex contractions of the right POOM STFs during maximal downgaze (effortful voluntary contractions of the GLIRM FTFs that enhanced ITM stretch) combined with maximal bilateral upper eyelid opening (effortful contractions of the LPSM and GLSRM FTFs that further enhanced STM stretch). This demonstrated that the contractions were driven by additive overstimulation of STM mechanoreceptors rather than by ITM stretch alone. Because the rostral superior colliculus (RSC) is the core structure for microsaccade generation [12,13] via the RLC [14,15], and elevated arousal is known to increase microsaccadic velocity [16], we hypothesized that enhanced STM mechanoreceptor stretch not only raises arousal [3-6] but also accelerates the frequency and velocity of microsaccades in LPSM, GLSRM, and GLIRM FTFs via the MTN, RLC, and RSC. The accelerated microsaccades would continually restretch STM or ITM mechanoreceptors, thereby inducing clonic reflex contractions of LPSM or POOM STFs that manifest as frequent blinking (FB). To test this hypothesis, we examined two patients with bilateral disinsertions of the levator aponeurosis [17] and LER [11]. Because their STM and ITM mechanoreceptors were sensitized, effortful eyelid opening with voluntary contractions of the LPSM, GLSRM, and GLIRM FTFs was expected to enhance STM and ITM stretch and provoke FB. We modulated STM and ITM stretch by loading a 3 g weight on the upper eyelid and by digitally elevating or pulling down the lower eyelid, then quantified blink dynamics with video analysis.

## Case presentation

Written informed consent was obtained from both patients for the publication of these case reports and accompanying images/videos.

Case assessment and manipulation protocol

All clinical manipulations and video evaluations were conducted under standardized room illumination in the primary gaze and upgaze positions. Eyelid movement dynamics and blink kinetics were evaluated preoperatively and postoperatively (at two weeks and six months).

Mechanical Loading

A standardized 3-g metal weight was temporarily adhered to the unilateral upper eyelid margin to increase the mechanical load and enhance STM mechanoreceptor stretch during voluntary eyelid retraction.

Digital Manipulation

The lower eyelid margin was digitally elevated (to stretch the ITM mechanoreceptors) or digitally pulled down (to slacken the ITM).

Video Observation

Blink frequency and velocity were assessed using clinical video recordings to infer the microsaccades of the LPSM, GLSRM, and GLIRM FTFs. Because microsaccades exhibit a strong linear relationship between their peak velocity and amplitude [7], we hypothesized that the speed of eyelid closure during a blink could be determined by measuring the amplitude of the eyelid movement. Notably, ocular microsaccades were not measured directly via electrophysiology or search coils but were conceptually inferred as putative drivers of the observed eyelid dynamics.

Case 1

A 28-year-old woman presented with severe bilateral eye strain and occasional eye twitching. Medical history was unremarkable for systemic neurological or neuromuscular disorders. Family history was negative for movement disorders or blepharospasm. Past treatment included artificial tears for suspected dry-eye syndrome, which provided no relief. Detailed history revealed 10 years of daily soft contact-lens wear [17] and 13 years of wearing tight goggles for competitive swimming, the latter presumed to have strongly disinserted her levator aponeuroses and LERs from the tarsi [11].

Preoperatively, in primary gaze, digitally elevating the unilateral lower eyelid margin increased bilateral FB in a degree-dependent manner with ipsilateral dominance (Video 1). To confirm tarsal disinsertion and enhance STM stretch, a 3 g weight was loaded onto the unilateral upper eyelid margin (Videos 2, 3). Primary gaze with the weight loaded caused ipsilateral upper eyelid droop and markedly increased FB frequency and velocity (Videos 2, 3). Upgaze further accelerated FB dynamics. Digitally pulling down the ipsilateral lower eyelid ceased (Video 2) or decreased (Video 3) FB, whereas digitally elevating the lower eyelid accelerated FB frequency and velocity.

**Video 1:** Preoperative video of Case 1: digital elevation of the unilateral lower eyelid margin in primary gaze

**Video 2:** Preoperative video of Case 1: eyelid manipulation (pulling down vs. elevating) with a 3 g weight loaded on left upper eyelid

**Video 3:** Preoperative video of Case 1: eyelid manipulation with a 3 g weight loaded on right upper eyelid

The patient underwent bilateral levator aponeurosis reinsertion onto the tarsal plates using three 6-0 nonabsorbable sutures combined with partial lateral-horn release to reduce mechanical strain on the STM (Figure 1).

**Figure 1:**
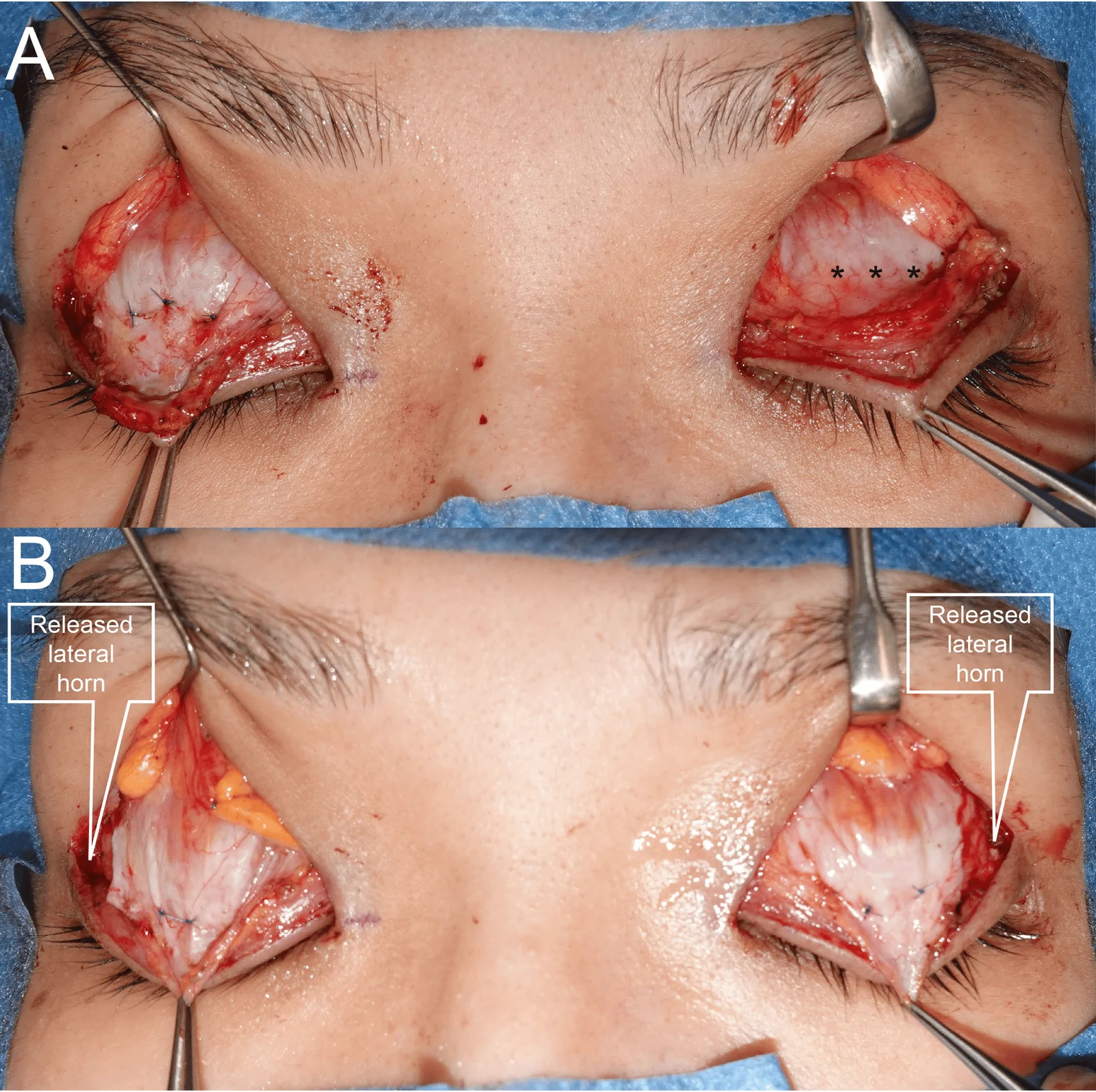
Intraoperative surgical findings of Case 1 (A) Reinsertion of the right levator aponeurosis to the tarsal plate using three 6-0 nonabsorbable sutures. Downward traction on the lid margins demonstrates severe disinsertion of the left levator aponeurosis from the tarsus with significant slippage. Asterisks (***) indicate the distal margin of the disinserted aponeurosis. (B) Bilateral aponeuroses successfully reinserted into the tarsal plates. The lateral horns were partially released to facilitate physiologic stretching of the STM during eyelid opening STM: superior tarsal muscle

At two weeks postoperatively, in primary gaze, digitally elevating the unilateral lower eyelid margin failed to provoke FB (Video 4). Loading a 3 g weight no longer interfered with upper eyelid retraction (Figure 2). At six months postoperatively, primary gaze with weight loading and digital lower eyelid elevation narrowed the palpebral fissure but produced no involuntary FB (Videos 5, 6). Eye strain was fully resolved.

**Video 4:** Two-week postoperative video of Case 1: digital lower eyelid elevation in primary gaze

**Figure 2:**
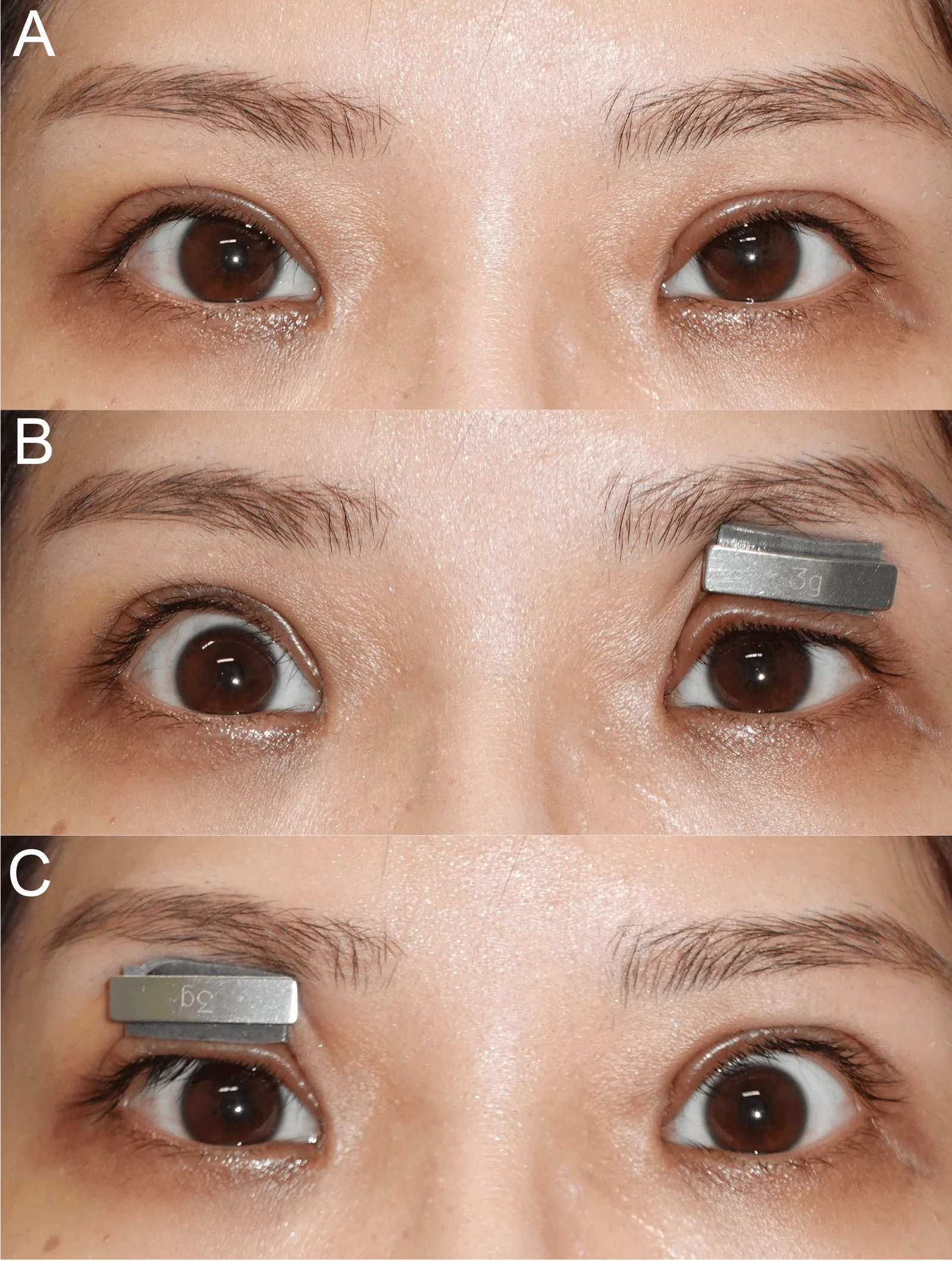
Two-week postoperative clinical photographs of Case 1 (A) Primary gaze. (B) Left upper eyelid 3 g weight loading demonstrating maintained eyelid retraction. (C) Right upper eyelid 3 g weight loading demonstrating maintained eyelid retraction

**Video 5:** Six-month postoperative video of Case 1: left weight loading and lower eyelid manipulation

**Video 6:** Six-month postoperative video of Case 1: right weight loading and lower eyelid manipulation

Case 2

A 46-year-old woman presented with chronic eye strain. Medical history was negative for neurological conditions, stroke, or movement disorders. Family history was noncontributory. History was notable for 28 years of daily contact-lens wear and frequent eyedrop use, presumed to have mildly disinserted her levator aponeuroses and LERs [11,17].

Preoperatively, a 3 g weight loaded onto the unilateral upper eyelid margin (Videos 7, 8) caused ipsilateral upper eyelid droop and bilateral narrowing of the palpebral fissures with ipsilateral dominance, without inducing typical FB. Upgaze increased fissure narrowing without inducing typical FB. Digitally pulling down the ipsilateral lower eyelid decreased fissure narrowing, whereas digitally elevating the unilateral lower eyelid bilaterally accelerated FB frequency and velocity.

**Video 7:** Preoperative video of Case 2: left upper eyelid weight loading and lower eyelid manipulation

**Video 8:** Preoperative video of Case 2: right upper eyelid weight loading and lower eyelid manipulation

At two weeks after aponeurosis reinsertion (Videos 9, 10), loading a 3 g weight onto the unilateral upper eyelid margin no longer caused ipsilateral upper eyelid droop or bilateral fissure narrowing. Digitally elevating the unilateral lower eyelid neither narrowed the palpebral fissures nor induced FB. The combination of digital elevation and upgaze narrowed the fissures but did not induce FB.

**Video 9:** Two-week postoperative video of Case 2: left weight loading and lower eyelid manipulation

**Video 10:** Two-week postoperative video of Case 2: right weight loading and lower eyelid manipulation The double-sided tape with the weight attached is restricting the upper eyelid movements

## Discussion

In Case 1, preoperatively, digital elevation of the unilateral lower eyelid margin in primary gaze (enhancing ITM mechanoreceptor stretch) bilaterally increased FB frequency and velocity with ipsilateral dominance in a degree-dependent manner (Video 1), consistent with the pathways shown in Figure 3. Unilaterally enhanced ITM stretch increased reflexive contractions of the bilateral POOM STFs with ipsilateral dominance [11]. These contractions bilaterally antagonized upper eyelid opening and bilaterally enhanced STM mechanoreceptor stretch, increasing proprioceptive signals to the MTN [10]. The MTN excited the oculomotor neurons (OMNs) (inducing reflexive LPSM STF contractions) and the RLC via gap junctions (GJs) [10]. Elevated RLC output increased arousal [3-6] and enhanced RSC sensitivity via the RLC-RSC projection [14,15], generating inferred microsaccades of FTFs in the LPSM, GLSRM, and GLIRM [12,13]. In patients with tarsal aponeurosis disinsertion [17], enhanced STM stretch increased RSC excitation, inhibiting omnipause neurons (OPNs) and unleashing rostral interstitial nucleus of the medial longitudinal fasciculus (riMLF) burst generators to accelerate inferred microsaccades via the OMNs [18,19]. Accelerated LPSM/GLSRM FTF microsaccades continually restretched STM mechanoreceptors, driving clonic reflexive LPSM STF contractions for eyelid opening [1,2]. Concurrently, accelerated GLIRM FTF microsaccades continually restretched ITM mechanoreceptors, driving clonic reflexive POOM STF contractions for eyelid closing [4,11]. The resulting antagonizing co-contraction loop between eyelid-opening and eyelid-closing muscles, based on accelerated inferred microsaccades, appears to exacerbate reflexive FB [20].

**Figure 3:**
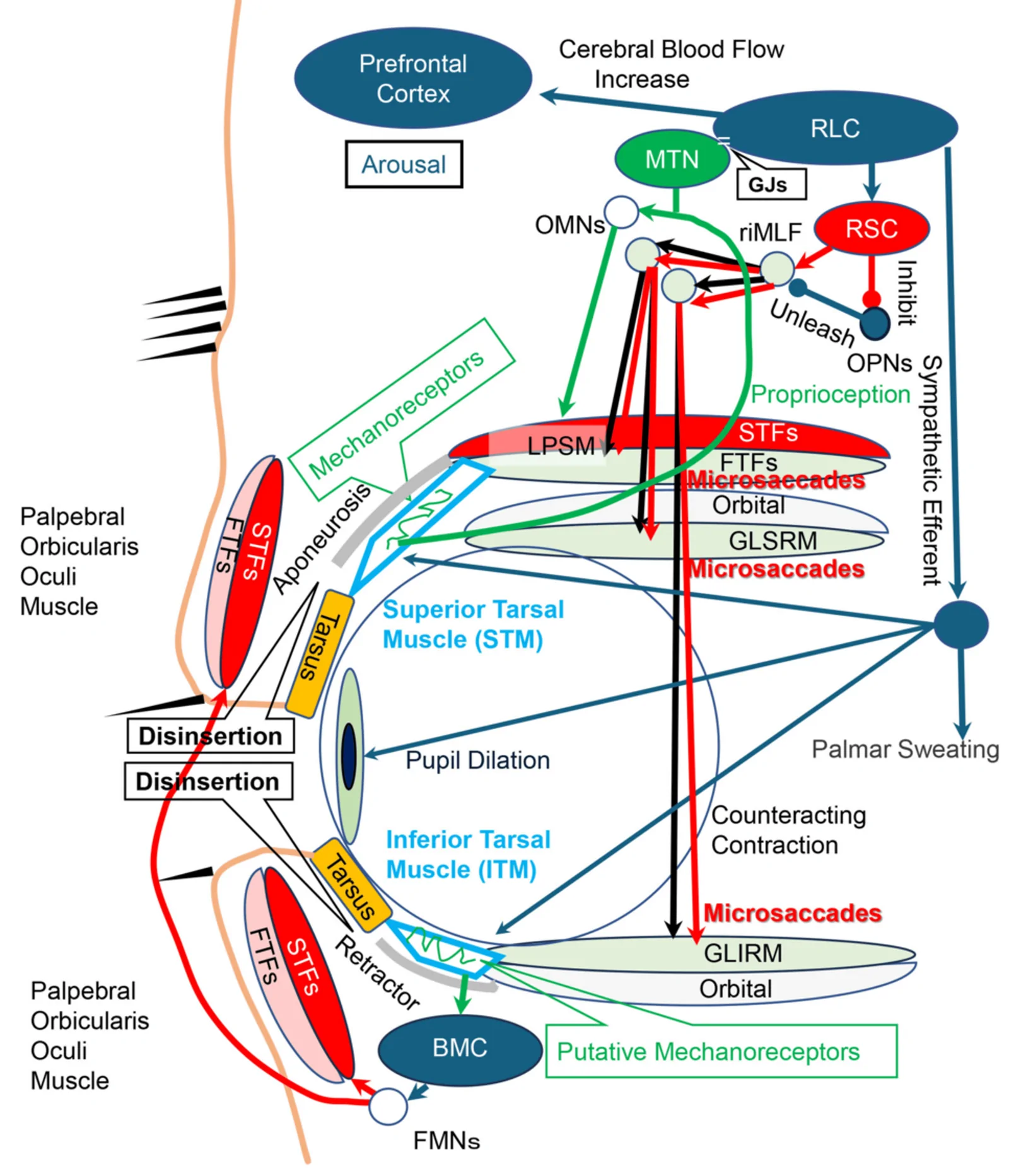
Proposed neuroanatomical mechanism governing FTF microsaccades and reflexive frequent blinking. Voluntary contractions of FTFs in the LPSM and GLSRM stretch STM mechanoreceptors, sending proprioceptive signals to primary neurons in the MTN. The MTN excites OMNs, inducing reflexive contractions of LPSM STFs and the RLC via GJs. Elevated RLC activity excites the prefrontal cortex for central arousal and the RSC for microsaccade generation. Enhanced RSC excitation inhibits OPNs, unleashing burst generators in the riMLF to accelerate inferred microsaccades of FTFs in the LPSM, GLSRM, and GLIRM via OMNs. Accelerated LPSM/GLSRM microsaccades continually restretch STM mechanoreceptors, driving clonic reflexive STF contractions of the LPSM for eyelid opening. Concurrently, accelerated GLIRM FTF microsaccades continually restretch ITM mechanoreceptors, driving clonic reflexive STF contractions of the POOM for eyelid closing via the BMC, including FMNs. The antagonizing clonic reflexive contractions exacerbate reflexive frequent blinking. Tarsal reinsertion of the disinserted levator aponeurosis reduces excessive baseline STM stretch BMC: brainstem motor center; FMN: facial motor neuron; FTF: fast-twitch fiber; GJ: gap junction; GLIRM: global layer of the inferior rectus muscle; GLSRM: global layer of the superior rectus muscle; ITM: inferior tarsal muscle; LPSM: levator palpebrae superioris muscle; MTN: mesencephalic trigeminal nucleus; OMN: oculomotor neuron; OPN: omnipause neuron; POOM: palpebral orbicularis oculi muscle; riMLF: rostral interstitial nucleus of the medial longitudinal fasciculus; RLC: rostral locus coeruleus; RSC: rostral superior colliculus; STF: slow-twitch fiber; STM: superior tarsal muscle Image credit: The authors Kiyoshi Matsuo and Ai Kaneko created the figure using Microsoft PowerPoint (Microsoft Corporation, Redmond, WA) based on [11]

Loading a 3 g weight onto the unilateral upper eyelid in primary gaze (Videos 2, 3) caused ipsilateral upper eyelid droop due to transmission failure between the LPSM muscle belly and the tarsus secondary to aponeurosis disinsertion [17]. This induced effortful bilateral upper and lower eyelid opening with increased voluntary contractions of the bilateral LPSM, GLSRM, and GLIRM FTFs, bilaterally enhancing both STM and ITM mechanoreceptor stretch. Enhanced STM stretch increased reflexive LPSM STF contractions via the MTN [1,2] and accelerated inferred microsaccades of the bilateral LPSM, GLSRM, and GLIRM FTFs via the MTN-RLC-RSC-OPN-riMLF-OMN pathway (Figure 3). Accelerated LPSM/GLSRM microsaccades continually enhanced bilateral STM stretch, driving clonic reflexive LPSM STF contractions with ipsilateral dominance. Accelerated GLIRM microsaccades continually enhanced bilateral ITM stretch, driving clonic reflexive POOM STF contractions with ipsilateral dominance. The antagonizing clonic contractions bilaterally exacerbated reflexive FB. Upgaze further exacerbated FB through additional acceleration of inferred microsaccades. Digitally pulling down the ipsilateral lower eyelid slackened the ipsilateral ITM, abolished bilateral POOM reflex contractions [11], reduced bilateral STM stretch below the threshold for accelerating microsaccades, and thereby alleviated FB. Conversely, digitally elevating the ipsilateral lower eyelid enhanced ITM stretch, increased bilateral POOM reflex contractions, further enhanced STM stretch, accelerated microsaccades, and exacerbated FB.

Surgical aponeurosis reinsertion restored transmission between the LPSM muscle belly and the tarsus and reduced baseline STM stretch. Consequently, at two weeks and six months postoperatively, digital lower eyelid elevation and upper eyelid weight loading induced POOM reflex contractions and effortful eyelid opening but no longer accelerated inferred microsaccades sufficiently to produce FB (Videos 4-6; Figure 3).

In Case 2, preoperatively (Videos 7, 8), unilateral upper eyelid weight loading caused ipsilateral droop and bilateral fissure narrowing with ipsilateral dominance without typical FB. Increased LPSM and GLSRM FTF contractions did not stretch the STM sufficiently to accelerate microsaccades; however, increased GLIRM contractions stretched the ITM, inducing bilateral POOM STF contractions and fissure narrowing. Upgaze produced small reflexive FB by further enhancing STM stretch enough to generate limited inferred microsaccades. The combination of weight loading and lower eyelid elevation, in a degree-dependent manner, induced clear FB by accelerating microsaccades sufficiently to drive antagonizing LPSM and POOM STF contractions. At two weeks postoperatively (Videos 9, 10), the same maneuvers narrowed the ipsilateral fissure via POOM contractions but no longer induced FB because the surgically stabilized STM could not be stretched enough to accelerate microsaccades.

Neurologically, voluntary contractions of LPSM, GLSRM, and GLIRM FTFs [8] stretch STM [9] and ITM [11] mechanoreceptors. STM stretch activates MTN trigeminal proprioceptive neurons (Figure 3) [10], inducing reflexive LPSM STF contractions [1,2] and exciting the RLC via GJs [10]. The RLC modulates both the prefrontal cortex (arousal) [3-6] and the RSC (microsaccade generation) [12,13]. Heightened RLC output therefore accelerates both systemic arousal and inferred microsaccades in the LPSM, GLSRM, and GLIRM FTFs via the MTN-RSC-riMLF-OMN pathway [7,12-16]. Inferred microsaccades in the GLSRM and GLIRM prevent visual fading, while those in the LPSM and GLSRM prevent proprioceptive fading by continually restretching STM mechanoreceptors, thereby involuntarily maintaining arousal and keeping the upper eyelids open. Concurrently, GLIRM microsaccades stretch ITM mechanoreceptors, inducing POOM STF contractions that adhere the eyelids to the globe [11]. Under normal conditions, these microsaccades do not produce visible FB. In patients with tarsal disinsertions, however, forceful eyelid opening hyperactivates the loop: enhanced STM stretch strongly excites the RSC via the MTN-RLC axis, inhibits OPNs, and unleashes riMLF burst generators, accelerating FTF microsaccades across the LPSM, GLSRM, and GLIRM [18,19]. The resulting continual restretching of tarsal mechanoreceptors drives persistent antagonizing co-contractions of LPSM and POOM STFs, exacerbating reflexive FB [20]. Enhanced ITM stretch further increases POOM resistance to upper eyelid opening, amplifying STM stretch beyond the effect of a 3 g weight. This self-sustaining cycle may progressively scale up arousal together with oculomotor and facial-motor hyperactivity, offering novel pathophysiological insights into focal dystonias such as blepharospasm and Meige syndrome [3-6].

This report is limited to two descriptive clinical cases. Microsaccades and specific extraocular-muscle fiber contractions were not directly measured with objective electrophysiological techniques (e.g., electromyography) or high-speed eye-tracking/scleral search-coil systems. Microsaccadic dynamics were inferred from video analysis and established neuroanatomical models. Clinical eyelid manipulations and manual evaluations carry a potential for observer bias. Future prospective studies incorporating simultaneous high-speed video-oculography, eyelid electromyography, and central neuroimaging in larger cohorts are required to validate the proposed neural pathways.

## Conclusions

Excessive mechanical stretching of STM mechanoreceptors in patients with tarsal disinsertions is associated with accelerated reflexive FB. We propose that enhanced STM mechanoreceptor stretch accelerates inferred FTF microsaccades across the LPSM, GLSRM, and GLIRM via the MTN-RLC-RSC-OPN-riMLF-OMN axis. Accelerated microsaccades continually restretch tarsal mechanoreceptors, establishing an antagonizing coactivation loop between eyelid-opening (LPSM) and eyelid-closing (POOM) STFs. Surgical reinsertion of the levator aponeurosis reduces mechanical strain, restores reflex homeostasis, and eliminates FB.
